# Does minimally invasive transforaminal lumbar interbody fusion (MIS-TLIF) influence functional outcomes and spinopelvic parameters in isthmic spondylolisthesis?

**DOI:** 10.1186/s13018-022-03144-y

**Published:** 2022-05-15

**Authors:** Elsayed Mohamed Selim Ali, Tarek Abdelsamad El-Hewala, Amr Mohamed Eladawy, Reda Ali Sheta

**Affiliations:** 1 Orthopedic Department, Zagazig University Hospital, Qumia, Nour Hoda Street, Zagazig, Egypt; 2 Al-Ahrar Specialist Hospital, 1st Talaat Harb Street from El Salm Street, Beside Sednawey Hospital, Zagazig, Al-Sharkia, 44759 Egypt

**Keywords:** Isthmic spondylolisthesis, Minimal invasive, Spinopelvic harmony, Spondylolysis

## Abstract

**Purpose:**

We assessed the efficacy of minimally invasive transforaminal lumbar interbody fusion (MIS-TLIF) in patients with low-grade isthmic spondylolisthesis.

**Methods:**

We included 24 symptomatic patients who underwent MIS-TLIF between December 2017 and December 2020. Patients were followed up clinically by the Oswestry Disability Index (ODI) and Visual Analogue Scale (VAS) for back and VAS for leg pain, as well as radiological radiographs after 6 weeks, 6 months, and at final follow-up (at least 12 months). Measured parameters included C7 sagittal vertical axis (SVA), pelvic tilt (PT), pelvic incidence (PI), sacral slope (SS), Meyerding slip grades, lumbar lordosis (LL), L1–L4 angle, L4–S1 angle, and segmental lordosis (SL) of the affected segment. The mismatch between the PI and LL was also measured.

**Results:**

VAS for back, VAS for leg pain, and ODI significantly improved postoperatively (all *p* < 0.001). We observed significantly decreased mean values of PT and slip percentage and increased mean values of SS and LL (all *p* < 0.05). We observed a significant reduction in L1–L4 lordosis and a significant increase in L4–S1 lordosis. The final PT, SS, and LL (total and L1–L4) were significantly higher in group III patients (*n* = 15) than the values of group II patients (*n* = 9). None of the patients became unbalanced postoperative, and all patients had a normal matching between the PI and the LL postoperatively.

**Conclusions:**

MIS-TLIF is a safe procedure for managing low-grade isthmic spondylolisthesis with significant improvement in clinical and radiological outcomes. It can correct and maintain a proper spinopelvic alignment.

**Supplementary Information:**

The online version contains supplementary material available at 10.1186/s13018-022-03144-y.

## Introduction

Men have twice as many pars interarticularis defects as women; however, women are more likely to progress to spondylolisthesis. The overall incidence of isthmic spondylolisthesis is about 4–8% of the general population and occurs most often at L5–S1 level and L4–L5 level [1]. Patients with symptomatic isthmic spondylolisthesis present with back pain with or without leg pain and a variable degree of neurological affection.

According to the Spinal Deformity Study Group (SDSG), spondylolisthesis is classified into low and high grades based on the slip percentage (%). The low grade is further classified according to pelvic incidence (PI) into three types where the group I patients have a PI less than 45°, group II patients have a PI from 45° to 60°, and group III patients have a PI of more than 60° [2].

Fusion is the treatment of choice when the conservative management fails to relieve patients’ symptoms, and the posterior approach is the standard access for achieving fusion in such cases, where the interbody fusion shows better results than posterolateral fusion [3].

In spondylolisthesis, transforaminal interbody fusion (TLIF) aims to stabilize the spinal motion segment, decompress the neural structures, restore the disc space height, and correct the spinopelvic relationship by correcting the local kyphotic deformity in spondylolisthesis [4].

We conducted this study to evaluate the efficacy of the minimally invasive transforaminal lumbar interbody fusion (MIS-TLIF) in improving the functional outcome in patients with low-grade isthmic spondylolisthesis by correcting the local spinopelvic deformity [3–7].

## Materials and methods

### Study process and eligibility criteria

We conducted this prospective cohort study on 24 patients with low-grade isthmic spondylolisthesis with back with or without leg pain who underwent MIS-TLIF at the Orthopedic Department, Zagazig University Hospitals, during the period between December 2017 and December 2020. We excluded patients with any of the following conditions: (1) patients who had been treated for degenerative lumbar disease, (2) patients who had no degenerative etiologies such as trauma, tumor, or infection, (3) patients who had undergone previous fusion surgery, or (4) patients who had a site of pathology other than the lumbar spine by whole spine sagittal magnetic resonance imaging (MRI). All patients underwent a trial of non-operative conservative treatment, including medication, physical therapy, and pain block, for at least 3 months before surgery. The patients were recommended for a surgical procedure after the failure of the non-operative treatment, and all patients continued to have significant back and leg pain with a significant restriction of daily activities due to radicular or neurogenic claudication.

### Preoperative assessment

**Fig. 1:**
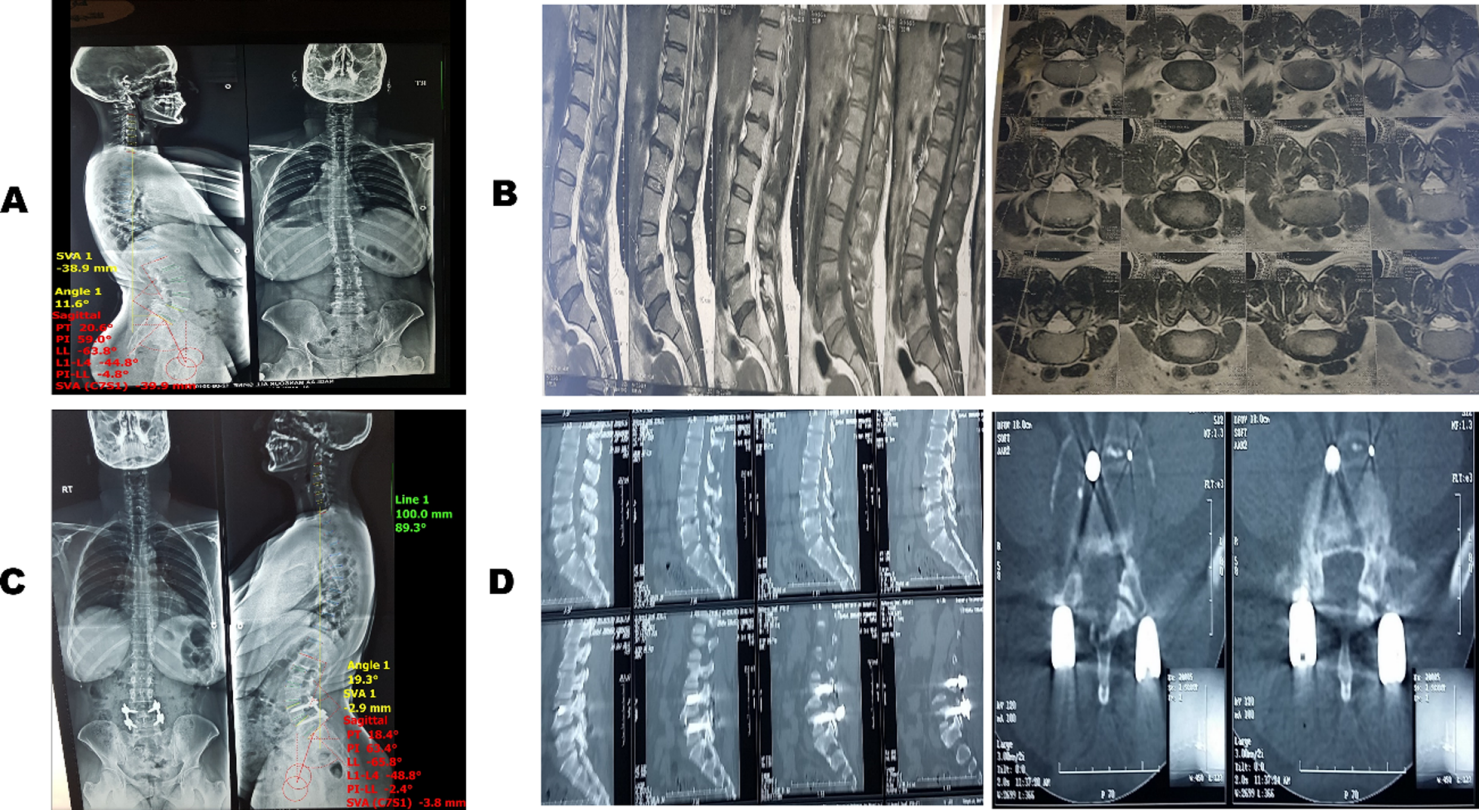
**a** Preoperative long-standing X-ray with spinopelvic parameters measurements, **b** preoperative MRI showing L4–5 isthmic spondylolisthesis, **c** postoperative long-standing X-ray with full correction and spinopelvic parameters measurements, and **d** postoperative follow-up CT at 1 year showing well union

Demographic data from all the patients, including age, sex, occupation, smoking, and body mass index (BMI), were collected. General, local, and neurological examination was performed on all patients. Before the operation, long-standing X-ray films from occiput to pelvis showing both femoral heads were taken (Fig. 1). Spinopelvic parameters were measured using Surgimap Spine. The measured parameters included: (a) C7 sagittal vertical axis (SVA): representing the distance between the center of C7 and the post point of the upper sacral endplate), (b) pelvic tilt (PT): representing the angle between a vertical line passing through the femoral head center and a line joining the center of the femoral head to the midpoint of the sacral endplate, (c) PI: representing the angle between a line perpendicular to the midpoint of the sacral plate and another line connecting the previous point to the center of the femoral axis, (d) sacral slope (SS): representing the angle between the S1 endplate and the horizontal plane and Meyerding slip grades, (e) lumbar lordosis (LL): *L1–L4 angle* representing the angle between the upper-end plate of L1 and the upper endplate of L4) and *L4–S1 angle* representing the angle between the upper-end plate of L4 and S1, and (f) segmental lordosis (SL) of the affected segment represents the angle between the upper endplate of the slipped vertebrae and upper endplate of the lower one.

All parameters were measured according to accepted protocols, as initially proposed by Legaye [5]. All included patients had dynamic films along with an MRI [6]. All radiologic parameters were evaluated using PACS software and a PACS workstation (Centricity 3.0, General Electric Medical System, Milwaukee, WI, USA). Oswestry Disability Index (ODI) and Visual Analogue Scale (VAS) scores for back and leg pain were obtained by all patients before surgery.

### Surgical technique (Fig. 2)

**Fig. 2:**
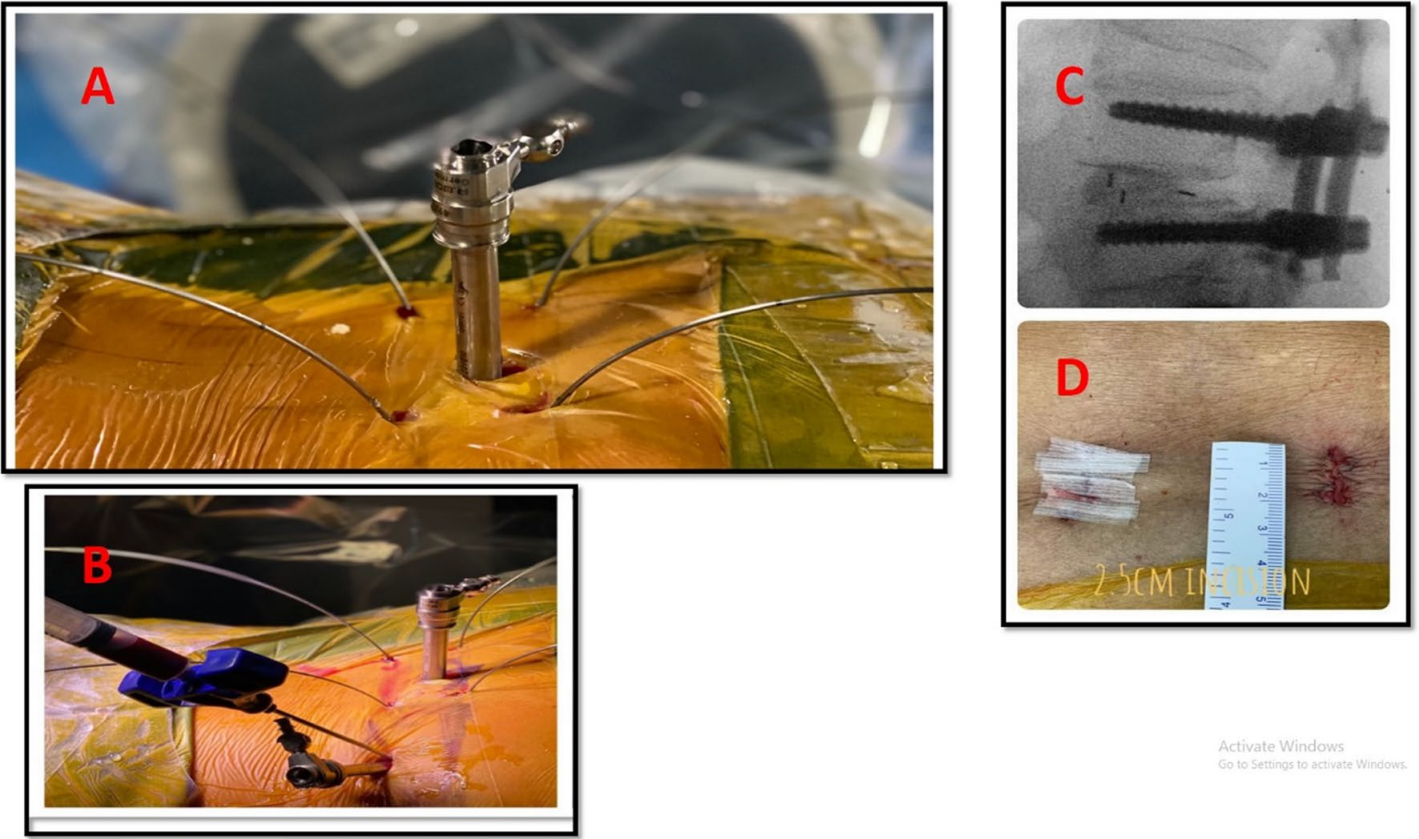
**A** An intraoperative photograph of the wound with the percutaneous screws’ towers, **B** under C-arm-guided insertion of screws in AP and lateral view, and **C** wound size after closure, **D** the wound size about 2.5 CM after closure

All patients were positioned prone on a radiolucent table. Four guide wires were inserted percutaneously into the pedicles through two paramedian skin incisions. On the non-symptomatic side, a facetectomy of the inferior process of the upper level and part of the superior facet of the lower level was done through the tubular system [2, 3]. This was followed by inserting the pedicle screws on that side [4]. Then, another facetectomy on the symptomatic side with the removal of the ligamentum flavum and more decompression was done. The disc space preparation with gradual distraction and release with sequential shavers was performed, followed by insertion of a single bullet-shaped polyether ether ketone (PEEK) interbody cage as anterior as possible [5]. The interbody cage was filled only with autologous bone chips, which were taken from the laminectomy [2, 3]. Finally, the pedicle screws on the symptomatic sides were inserted with the application of the rods on both sides. Compression was applied to the screws to maximize the segmental lordosis [5]. The posterolateral transverse process arthrodesis was not performed in this study. We documented the operative data, including the blood loss, radiation exposure, and operative time.

### Follow-up

Patients were followed up clinically by the ODI questionnaire and VAS for back and VAS for leg pain as well as radiologically by anteroposterior (AP) and lateral radiographs after 6 weeks, 6 months, and at final follow-up. At the final follow-up, long-standing X-ray films from occiput to pelvis showing both femoral heads were done to measure the spinopelvic parameters. A CT scan was done at the final follow-up to detect union. The modified Bridwell fusion criteria for the lumbar spine were used to assess fusion; grades I and II were considered satisfactory fusion.

### Statistical analysis

The Statistical Package for Social Science (SPSS) software version 20.0 was used for the statistical analysis. We presented the qualitative data as frequencies and relative percentages, while the quantitative data were presented as mean and standard deviation (SD) or median and range. Findings of the quantitative data were compared by using the Mann–Whitney U test, paired *t* test, Wilcoxon signed-rank test, and Friedman’s test, which were used as appropriate. The chi-square test and the Fisher’s exact probability were used to compare the qualitative data as appropriate. *P* values of less than 0.05 were considered statistically significant.

## Results

### Patient demographics and operative data

A total of 24 patients (20 female and 4 male), with a mean age of 40.42 years (SD = 4.65) and a mean BMI of 28.12 kg/m^2^ (SD = 7.82), were included. The majority of patients (*n* = 17; 70.8%) had spondylolisthesis at the L5–S1. All included patients had back and leg pain. The mean follow-up period was 19.42 months (SD = 6.26). The mean operative time was about 110.20 min (SD = 13.39), the mean radiation exposure was 3.79 min (SD = 0.83), and the mean amount of blood loss was 56.45 cc (SD = 14.63). None of our study participants required blood transfusion during the perioperative period. The patient demographics and operative data of the operated patients are shown in Table 1.

**Table 1:** Patient demographics and operative data among the operated patients (n = 24)

Patient demographics	
	n = 24
*Sex*	
Male	4 (16.7%)
Female	20 (83.3%)
*Age (years)*	
Mean ± SD	40.42 ± 4.65
Median (range)	42 (32–47)
*Body mass index (kg/m*^*2*^*)*	
Mean ± SD	28.12 ± 7.82
Median (range)	29 (19–44)
Comorbidity	
*HCV*	
Absent	21 (87.5%)
Present	3 (12.5%)
*Smoking*	
Smoker	1 (4.2%)
Non-smoker	23 (95.8%)
*Follow-up (months)*	
Mean ± SD	19.42 ± 6.26
Median (range)	16 (13–24)
*Level of spondylolisthesis*	
L5–S1	17 (70.8%)
L4–L5	7 (29.2%)
*Spinal deformity study group*	
Grade 2	9 (37.5%)
Grade 3	15 (62.5%)
Operative data	
*Radiation exposure (min.)*	
Mean ± SD	3.79 ± 0.83
Median (range)	4 (3–5)
*Blood loss (cc)*	
Mean ± SD	56.45 ± 14.63
Median (range)	52.50 (40–90)
*Operation duration (min.)*	
Mean ± SD	110.20 ± 13.39
Median (range)	110 (90–135)

### Functional outcome measures

We observed significantly improved functional outcome measures postoperatively (all *p* < 0.001). The VAS for back and VAS for leg pain significantly decreased from 8.42 (SD = 0.58) and 7.46 (SD = 0.98) to 2.17 (SD = 0.38) and 1.25 (SD = 0.44) at 6 months and 1.79 (SD = 0.88) and 1.00 (SD = 1.59) at the final follow-up, respectively. Moreover, the ODI significantly decreased from severe disability (52.21%; SD = 6.79) to mild disability 14.04% (SD = 2.35) at 6 months and continued until the final follow-up, as shown in Table 2.

**Table 2:** Functional outcome measures and post hoc test results

	Baseline	Postoperative			Test*	*p* value (Sig.)
		6 weeks	6 months	Final		
*Visual Analogue Scale (VAS) for back*						
Mean ± SD	8.42 ± 0.58	3.58 ± 0.65	2.17 ± 0.38	1.79 ± 0.88	62.074*	< 0.001 (HS)
Median (range)	8 (7–9)	3.5 (3–5)	2 (2–3)	1.5 (1–3)		
*Visual Analogue Scale (VAS) for leg*						
Mean ± SD	7.46 ± 0.98	3.33 ± 0.48	1.25 ± 0.44	1.00 ± 1.59	66.792*	< 0.001 (HS)
Median (range)	7 (6–9)	3 (3–4)	1 (1–2)	1 (0–8)		
*Oswestry Disability Index (ODI)%*						
Mean ± SD	52.21 ± 6.79	17.54 ± 1.25	14.04 ± 2.35	15.71 ± 9.95	59.484*	< 0.001 (HS)
Median (range)	51 (42–63)	18 (15–19)	14 (10–19)	14.5 (10–61)		

Post hoc test results						
	Preoperative versus 6 weeks	Preoperative versus 6 months	Preoperative versus final	6 weeks versus 6 months	6 weeks versus final	6 months versus final
*Visual Analogue Scale (VAS) for back*						
Test‡	− 4.451	− 4.373	− 4.323	− 4.092	− 4.011	− 1.806
*p* value (Sig.)	< 0.001 (HS)	< 0.001 (HS)	< 0.001 (HS)	< 0.001 (HS)	< 0.001(HS)	0.071
*Visual Analogue Scale (VAS) for Leg*						
Test‡	− 4.344	− 4.328	− 4.319	− 4.518	− 3.7	− 2.337
*p* value (Sig.)	< 0.001 (HS)	< 0.001 (HS)	< 0.001 (HS)	< 0.001 (HS)	< 0.001 (HS)	0.019
*Oswestry Disability Index (ODI)*						
Test‡	− 4.289	− 4.29	− 4.26	− 3.75	− 3.189	− 0.241
*p* value (Sig.)	< 0.001 (HS)	< 0.001 (HS)	< 0.001 (HS)	< 0.001 (HS)	0.001 (S)	0.809

^*^Friedman’s test was used, ‡ Wilcoxon signed-rank test, *p* < 0.05 is significant and Sig.: Significance

### Radiological outcome measures

The mean PI did not change throughout the follow-up (*p* = 0.196). We observed significantly decreased mean values of PT (21.07 versus 19.84, *p* < 0.001) and slip percentage (23.13 vs. 6.48, *p* < 0.001) as well as increased mean values of SS (40.72 vs. 41.98, *p* < 0.001) and LL (57.23 versus 57.94, *p* = 0.047). Significant improvement toward more normal values for PT and SS in relation to PI was observed postoperatively. There was an insignificant reduction in the mismatch from 10.33 (SD = 7.48) to 6.21 (SD = 2.85). We observed a significant reduction in L1–L4 lordosis (30.10 vs. 27.65, *p* = 0.006) and a significant increase in L4–S1 lordosis (35.07 vs. 38.88, *p* < 0.001). The mean SL significantly increased from 16.30 (SD = 6.52) to 20.70 (SD = 6.49), with a *p* value of < 0.001. The results of the radiological outcome measures are shown in Table 3 and Additional file 1: Table S1.

**Table 3:** Radiological outcome measures

	Baseline	Postoperative			Test	*p* value (Sig.)
		6 weeks	6 months	final		
*Pelvic incidence (PI)*						
Mean ± SD	61.83 ± 8.77	61.78 ± 8.88	61.61 ± 8.90	61.61 ± 8.99	1.654*	0.196
Median (range)	62.15 (50.2–77.9)	62.15 (50.3–78)	62.15 (49.7–78.1)	62.15 (49–78.1)		
*Pelvic tilt (PT)*						
Mean ± SD	21.07 ± 8.74	26.42 ± 5.99	21.12 ± 5.20	19.84 ± 4.20	36.620‡	< 0.001 (HS)
Median (range)	20.6 (8–32.9)	28.35 (15–35)	19.25 (13–31.5)	19.45 (12.3–27)		
*Sacral slope (SS)*						
Mean ± SD	40.72 ± 7.03	35.62 ± 6.01	40.69 ± 5.73	41.98 ± 6.19	22.410*	< 0.001 (HS)
Median (range)	40.70 (25.9–54)	34.90 (23–45.9)	40.90 (32.2–50)	42.6 (32.1–52.9)		
*Lumbar lordosis (LL)*						
Mean ± SD	57.23 ± 9.23	54.87 ± 6.88	57.78 ± 7.20	57.94 ± 6.83	7.213‡	0.047 (S)
Median (range)	56.35 (40–73)	57.15 (42–65.1)	58 (44.9–68.4)	59 (47.4–68.9)		
*Slip (%)*						
Mean ± SD	**23.13 ± 8.09**	5.13 ± 3.97	5.98 ± 4.71	6.48 ± 5.58	53.27‡	< 0.001 (HS)
Median (range)	23.5 (5–42)	4 (1–16)	4.5 (1.8–18)	4.5 (1.8–23)		
*Mismatch*						
Mean ± SD	10.33 ± 7.48	7.67 ± 4.90	6.31 ± 2.85	6.21 ± 2.85	4.513‡	0.211
Median (range)	8.55 (0–23.7)	7.20 (0.1–18)	7.30 (1–9.9)	7.10 (0.1–9.9)		
*L1–L4*						
Mean ± SD	30.10 ± 8.02	27.41 ± 7.21	27.88 ± 7.29	27.65 ± 6.56	12.443‡	0.006 (S)
Median (range)	30.80 (15.6–41)	28.95 (13.7–34.2)	28.80 (15.7–41.1)	27.50 (17.2–38.6)		
*L4–S1*						
Mean ± SD	35.07 ± 6.47	37.55 ± 5.09	37.92 ± 4.2	38.88 ± 4.24	12.602*	< 0.001 (HS)
Median (range)	34.15 (23.8–45)	38 (27.9–44.5)	38.50 (32–45)	39.15 (31.2–45.2)		
*Segmental lordosis (SL)*						
Mean ± SD	16.30 ± 6.52	22.23 ± 7.10	21.12 ± 6.50	20.70 ± 6.49	49.034‡	< 0.001(HS)
Median (range)	14.40 (4.2–30)	19 (14.8–38.9)	19.40 (13.9–38)	19.10 (13.9–37)		

^*^Repeated measures ANOVA test, ‡ Friedman's test

S: Significant(*P* < 0.05); HS: Highly significant (*P* < 0.01)

### Comparison according to the PI-LL mismatch

Based on the PI-LL mismatch, the operated patients were divided into two groups. The normal group included 14 patients with PI-LL equal to ±10, while the abnormal group included 10 patients with a PI-LL mismatch of more than 10. Compared to the baseline (preoperative) data, the final follow-up data showed significant changes in terms of ODI (*p* = 0.040), PT (*p* = 0.022), SS (*p* = 0.020), LL (*p* = 0.007), and SL (*p* = 0.002), as shown in Table 4.

**Table 4:** The comparison according to the mismatch between the PI and LL

Parameters	Mismatch		Test§	*p* value
	Normal (N = 14)	Abnormal (N = 10)		(Sig.)
*Δ Visual Analogue Scale (VAS) for back*				
Mean ± SD	6.71 ± 1.20	6.50 ± 1.17	− 0.426	0.670
Median (range)	7 (5–8)	6 (5–8)		
*Δ Visual Analogue Scale (VAS) for leg*				
Mean ± SD	6.28 ± 1.89	6.70 ± 0.82	− 0.333	0.739
Median (range)	6 (1–8)	7 (5–8)		
*Δ Oswestry Disability Index (ODI)*				
Mean ± SD	32.64 ± 11.55	41.90 ± 8.69	− 2.056	0.040 (S)
Median (range)	36.50 (− 3 to 47)	45.50 (27–51)		
*Δ Pelvic incidence (PI)*				
Mean ± SD	− 0.34 ± 0.86	− 0.03 ± 0.26	− 0.709	0.478
Median (range)	0 (− 2.80 to 0.60)	0 (- 0.40 to 0.30)		
*Δ Pelvic tilt (PT)*				
Mean ± SD	0.88 ± 4.30	− 4.19 ± 6.43	− 2.288	0.022 (S)
Median (range)	1.20 (− 5.10 to 5.70)	− 5.90 (− 10.70 to 9)		
*Δ Sacral slope (SS)*				
Mean ± SD	− 1.05 ± 4.46	4.49 ± 6.49	− 2.317	0.020 (S)
Median (range)	− 1.15 (− 6.50 to 5.10)	7.10 (− 9 to 10.80)		
*Δ Lumbar lordosis (LL)*				
Mean ± SD	− 3.60 ± 5.05	6.73 ± 9.93	− 2.698	0.007(S)
Median (range)	− 2.25 (− 12.80 to 4)	9.80 (− 13 to 14.60)		
*ΔL1–L4*				
Mean ± SD	− 2.17 ± 5.20	− 2.82 ± 8.10	− 0.293	0.769
Median (range)	− 3.15 (− 11.20 to 7)	− 5.05 (− 12.30 to 7.80)		
*ΔL4–S1*				
Mean ± SD	2.75 ± 4.19	5.31 ± 4.04	− 1.642	0.101
Median (range)	2.95 (− 5 to 10.20)	7.90 (− 1.60 to 9)		
*Δ Segmental lordosis (SL)*				
Mean ± SD	2.34 ± 2.99	7.26 ± 3.44	− 3.165	0.002 (S)
Median (range)	3.15 (− 6.20 to 5.80)	6.55 (0.80–12.20)		

Δ VAS: VAS baseline–VAS final, Δ ODI: ODI baseline–ODI final, Δ PI: PI at final–PI baseline, Δ PT: PT final–PT baseline, Δ SS: SS final–SS baseline, Δ LL: LL final–LL baseline, Δ L1–L4: L1–L4 final–L1–L4 baseline, Δ L4–S1: L4–S1 final–L4–S1 baseline, Δ SL: SL final–SL baseline, § Mann–Whitney *U* test., *p* < 0.05 is significant and Sig.: Significance

### Comparison according to the SDSG grade

Based on the SDSG grade, the patients were classified into two groups. Nine patients had SDSG grade II (PI < 60), while 15 patients had SDSG grade III (PI > 60). The comparison of the baseline (preoperative) data and the final follow-up data revealed a significant change in terms of PT (− 2.79, SD = 5.66 in SDSG grade III subgroup versus 1.37 SD = 5.20 in SDSG grade II subgroup, *p* = 0.045). We observed no significant differences in terms of SS (*p* = 0.068), LL (*p* = 0.612), mismatch (*p* = 0.160), L1–L4 lordosis (*p* = 0.244), L4–S1 lordosis (*p* = 0.355), and SL (*p* = 0.136), Additional file 1: Table S2. Compared to the baseline (preoperative) data, the data at 12 months showed significant changes in terms of PT, SS, LL, and L1–L4 lordosis in the SDSG III group over the other group (all *p* < 0.05); however, the difference was not significant in terms of VAS for back, VAS for leg pain, ODI, mismatch, L4–S1 lordosis, and SL (all *p* > 0.05) (Table 5).

**Table 5:** Comparison according to the SDSG grade at 12 months

Parameters	SDSG		Test§	*p* value
	SDSG 2 (*N* = 9)	SDSG 3 (*N* = 15)		(Sig.)
*Visual Analogue Scale (VAS) for back*				
Mean ± SD	1.44 ± 0.72	2 ± 0.92	− 1.462	0.144
Median (range)	1 (1–3)	2 (1–3)		
*Visual Analogue Scale (VAS) for leg*				
Mean ± SD	0.77 ± 0.44	1.13 ± 1.99	− 0.341	0.733
Median (range)	1 (0–1)	1 (0–8)		
*Oswestry Disability Index (ODI)*				
Mean ± SD	13.78 ± 2.108	16.87 ± 12.495	− 0.272	0.786
Median (range)	14 (11–17)	15 (10–61)		
*Pelvic tilt (PT)*				
Mean ± SD	17.02 ± 2.99	21.53 ± 3.95	− 2.534	0.011
Median (range)	16 (12.30–22.20)	21.90 (14–27)		(S)
*Sacral slope (SS)*				
Mean ± SD	35.4778 ± 2.28844	45.8733 ± 4.06966	− 8.006	< 0.001
Median (range)	35.8(32.1–40)	45 (39.7–52.9)		(HS)
*Lumbar lordosis (LL)*				
Mean ± SD	52.46 ± 4.95	61.22 ± 5.64	− 3.139	0.002
Median (range)	50 (47.40–59)	60 (50.80–68.90)		(S)
*Mismatch*				
Mean ± SD	5.22 ± 3.31	6.80 ± 2.76	− 1.283	0.2
Median (range)	4.60 (1.60–9.50)	7.20 (0.10–9.90)		
*L1–L4*				
Mean ± SD	23 ± 6.09	30.4 ± 5.32	− 2.419	0.016
Median (range)	20 (17– 32.2)	29.8 (22.3–38.6)		(S)
*L4–S1*				
Mean ± SD	37.44 ± 3.19	39.74 ± 4.64	− 1.404	0.16
Median (range)	38.10 (33.70–43.30)	41.90 (31.20–45.20)		
*Segmental lordosis (SL)*				
Mean ± SD	20.62 ± 3.01	20.74 ± 8	− 1.343	0.179
Median (range)	21.90 (14.60–23.60)	17.30 (13.90–37)		

^§^ Mann–Whitney *U* test, *p* < 0.05 is significant. Sig.: Significance

### Fusion rate and complications

The SVA range improved from (− 65.1 to 110 mm) to (− 29 to 35 mm). All patients had normal balance postoperatively. The fusion rate was 95.8%; only one patient showed nonunion due to cage subsidence (Additional file 2: Fig. S1). One patient had a small dural tear not reaching the arachnoid, which was managed conservatively. During the follow-up period, only 2 patients showed backward cage migration which was detected radiologically, with no neurological insult till the end of the study (Additional file 3: Fig. S2 and Additional file 4: Fig. S3).

## Discussion

Symptomatic patients with spondylolisthesis who experienced a failure of the conservative treatment usually need surgical intervention. Correcting the spinopelvic parameters has become a necessary goal in spondylolisthesis surgery [4]. We aimed to assess the role of MIS-TLIF in improving the functional outcome in patients with low-grade isthmic spondylolisthesis.

VAS and ODI are valuable indicators of the functional outcome in patients with lumbar spine problems, and their improvement indicates a successful treatment [7]. In our study, the functional outcome measures significantly improved after surgery. VAS for back and leg pain scores significantly decreased during the follow-up (all *p* < 0.05). We observed significantly decreased ODI scores from severe disability (52.21%) to mild disability (14.04%) at 6 months and continued until the final follow-up. Our findings are in line with the finding of Park P and Foley KT [8]. In their study, significant improvements in ODI and VAS scores were documented. The postoperative means values of VAS for leg pain and VAS for back pain scores decreased from 65 and 52 to 8 and 15, while the postoperative mean ODI score decreased from 55% (moderate disability) to 16% (minimal disability). Another study [9] studied the instrumented IS reduction for 12 patients treated by MIS-TLIF. The spondylolisthesis reduction was reported to be 95%, and the surgical outcomes were “excellent” and “good” in 8 and in 4 patients, with no documented neurologic complications. Our study showed early improvement in clinical outcomes through early ambulation, reduced hospital stay, and early return to work. This is in line with the findings of Parker et al., who investigated the effect of the MIS technique on return to work and narcotic use following TLIF surgery [10].

Spondylolisthesis reduction may help re-establish a correct balance of the spinal column by correcting the lumbosacral kyphosis, resulting in a lower risk of degenerative evolution of adjacent segments and improving the altered biomechanics of the spine. In the current study, the excellent slip correction was achieved as an improvement in slip percentage from 23.13% to 6.48%. This was achieved with the help of positional reduction through maintaining intraoperative physiologic lordosis with proper hip extension. A bilateral facetectomy with direct decompression of both foramina was utilized to complete release and improve the slip correction. Reducing the lumbosacral kyphosis may improve the biomechanical environment for a fusion by converting the shear forces to compressive forces [11].

PI is the mathematical summation of the two parameters, PT and SS, the SS is two-thirds of the PI, and the PT is within the other one-third. It is always assumed that keeping this normal relationship between them will keep the pelvis balanced [12]. A previous study on 214 patients with L5-S1 spondylolisthesis documented significantly higher PI than patients without spondylolisthesis. A similar study demonstrated that patients with low-grade lumbosacral spondylolisthesis had significantly higher PI than the control population (65.5 vs. 51.9) [13, 14]. Patients in our study had similar values; the mean value for PI was 61.83 (SD = 8.77), and the mean value of PI did not change during the follow-up (*p* = 0.196).

There was a significant decrease in the PT value from 21.07 to 19.84 in the final follow-up. However, the patients showed an initial increase in PT postoperatively (26.42, SD = 5.99) at 6 weeks, which then decreased. This may be due to the relief of hamstring spasms as compensation for the slippage. The improvement and maintenance of PT in physiological ranges might be one of the reasons why MIS-TLIF could improve the low back pain in isthmic spondylolisthesis. Aoki et al. [15] encourage surgeons to achieve sagittal spinopelvic alignment carefully and avoid postoperative PI-LL mismatch. PT reduction should increase the adaptive capacities of patients with lumbar pathologies. Moreover, its reduction is associated with less pain; this explains the improvement in ODI and VAS scores with PT reduction in our study [15].

We observed significantly increased mean values of SS and LL (*p* < 0.001 and *p* = 0.047) and significant postoperative improvement toward more normal values for PT and SS in relation to PI. Similar to our study, Martiniani et al. documented significantly decreased PT values from 41 to 30 and increased SS from 36 to 47 with fusion [16].

It is challenging to estimate the appropriate SL in patients with spondylolisthesis. Lauber et al. [17] observed an improvement in the mean slip percentage and the disc height with no increase in SL in 39 patients with spondylolisthesis undergoing the TLIF procedure. Also, Kwon et al. [3] addressed the slip reduction by performing TLIF on a group of 30 patients with isthmic spondylolisthesis; however, they were unable to restore the focal lordosis at the instrumented level. In contrast to previous studies, we observed improved SL from 16.30 to 20.70, and the lower lumbar (L4–S1) lordosis improved from 35.07 to 38.88. These results are comparable to the results of Mourad Olud-Slimane et al., who reported an 8.1° SL increase, and Galla et al., who reported a 5.7° SL increase [18, 19].

In our study, the postoperative LL significantly increased from 57.23 to 57.94. These changes were assumed to be associated with preserving the posterior tension band created by the posterior ligament complex, less injury to the paraspinal musculature by using the tubular system, and compressing the pedicle screws posteriorly against the anteriorly located interbody cage. The patients were divided into normal PI-LL match and PI-LL mismatch groups according to the PI-LL mismatch. Compared to the preoperative data, we observed significant changes in terms of ODI, PT, SS, LL, and SL LL between both groups (All *p* < 0.05). The mismatched group had a more preoperative disability and a more negative impact on health-related quality of life (HRQoL) than the normal group. This explains the significant postoperative improvement in the functional outcome and the radiological parameters in the mismatched group compared to the normal PI-LL match group. The rate of improvement in ODI was 41.90 (SD = 8.69) in the mismatched group compared to 32.64 (SD = 11.55) in the normal group. Similar improvements were noticed in the correction rate of PT, SS, LL, and segmental lordosis, with a significant difference between both groups where the mismatched group showed significant correction. Merrill et al. [20] support the concept that sagittal balance involves multiple parameters (including PT and PI-LL mismatch). When there was a normal SVA and PI-LL was > 10, patients could improve global sagittal alignment by a compensatory increase in PT to maintain normal SVA. While this compensation improves SVA, a persistent PI-LL mismatch and a high PT may predispose severe disability in those patients. They also assumed that PT must not exceed 20°–22° [20].

All patients in the current study had low-grade spondylolisthesis; of them, 15 patients had grade III SDSG, while the remaining nine patients had grade II SDSG. The final PT, SS, and LL (total and L1–L4) were significantly higher in group III patients than the values of group II patients. When comparing the rate of change occurring in each parameter between the preoperative and final postoperative values, there was a statistically insignificant difference between both groups except for the PT, which showed a higher rate of change in group III over group II (*p* = 0.045). This indicates that MIS-TLIF could correct the retroversion compensatory mechanism of the pelvis by changing the PT significantly to restore the normal orientation of the pelvis.

In the current study, MIS-TLIF in this patient series resulted in restoring and/or maintaining the spinopelvic harmony, correcting the focal kyphotic deformity, and restoring the SVA normal values. The range of SVA improved from a range of (− 65.1 to 110 mm) to (− 29 to 35 mm). Neglecting a high SVA and mismatched PI-LL can lead to worse surgical outcomes. The risk is high in patients with a high PI; because they attempt to maintain a tolerable upright posture by having more LL and increasing the PT by retroverting the pelvis. These compensatory mechanisms require more energy consumption; they lead to severe pain and impaired HRQoL [21].

The correlations between spinopelvic parameters and HRQoL scores in patients with adult spinal deformity were studied in a previous study. The authors stated that PT, PI-LL, and SVA could predict the disability and guide patient assessment for good decision-making. Patients with values of PT of 22° or more, SVA of 47 mm or more, and PI − LL of 11° or more were reported to be more likely to have a negative impact on the HRQoL [21]. None of the patients became unbalanced postoperative, and all patients had a normal matching between the PI and the LL postoperatively. Only 2 cases had preoperative abnormal SVA, which were rectified thereafter.

Our study has some limitations. First, the relatively small number of cases choosing only the isthmic cases may limit the generalization of the study findings. Second, the follow-up periods were not long enough to assess the long-term efficacy. Therefore, future studies that include large number of patients and long-term follow-up periods are warranted to support these findings.

## Conclusion

MIS surgeries have been accused of being incapable of improving the outcomes of open surgeries. On the contrary, this study showed that the MIS-TLIF improved the functional outcome (VAS back pain, VAS leg pain, and ODI) at the final follow-up. Even after classifying them according to SDSG, there was an insignificant difference between the final functional outcomes in both groups. In conclusion, MIS-TLIF is a reliable procedure for managing low-grade isthmic spondylolisthesis with significant improvement in clinical and radiological outcomes. It can correct and maintain a proper spinopelvic alignment.

## Supplementary Information

**Additional file 1.** Radiological outcome measures.

**Additional file 2: Figure S1** (case 2) shows (a) preoperative long-standing X-ray with spinopelvic parameters measurements, (b) preoperative MRI showing L5–S1 isthmic spondylolisthesis, (c) postoperative long-standing X-ray with spinopelvic parameters measurements, and (d) postoperative follow-up at 1-year follow-up CT shows nonunion.

**Additional file 3: Figure S2** (case 3) shows (a) preoperative long-standing X-ray with spinopelvic parameters measurements, (b) preoperative MRI showing L5–S1 isthmic spondylolisthesis, (c) postoperative long-standing X-ray with spinopelvic parameters measurements, and (d) postoperative follow-up CT shows backward migrations of the cage.

**Additional file 4: Figure S3** (case 3) shows under C-arm-guided insertion of screws in AP and lateral view, intraoperative photograph of the wound and the tube retractor tube, wound size after closure.

## Abbreviations

TLIF: Transforaminal lumbar interbody fusion
MIS-TLIF: Minimally invasive transforaminal lumbar interbody fusion
IS: Isthmic spondylolisthesis
DS: Degenerative spondylolisthesis
VAS: Visual Analogue Scale
ODI: Oswestry Disability Index
PI: Pelvic incidence
PT: Pelvic tilt
SS: Sacral slope
