# Supplementary material for: Does minimally invasive transforaminal lumbar interbody fusion (MIS-TLIF) influence functional outcomes and spinopelvic parameters in isthmic spondylolisthesis?
Source: J Orthop Surg Res. 2022 May 15;17:272. doi: 10.1186/s13018-022-03144-y (PMC9107691; doi:10.1186/s13018-022-03144-y)
Supplement: Supplementary file 1 — Additional file 1. Radiological outcome measures. [file 13018_2022_3144_MOESM1_ESM.docx]

Appendix Table S1. The post hoc test results of radiological outcome measures

|  | Preoperative vs. 6 weeks | Preoperative vs. 6 months | Preoperative vs. final | 6 weeks vs. 6 months | 6 weeks vs. final | 6 months vs. final |
| --- | --- | --- | --- | --- | --- | --- |
| Pelvic Tilt (PT) | | | | | | |
| Test‡ | -4.031 | -0.257 | -0.743 | -4.108c | -4.289 | -3.009 |
| p-value (Sig.) | <0.001 (HS) | 0.797 | 0.457 | <0.001(HS) | <0.001 (HS) | 0.003 |
| Sacral Slope (SS) | | | | | | |
| Test* | 5.104 | 0.033 | -1.254 | -5.071 | -4.358 | -1.287 |
| p-value (Sig.) | <0.001 (HS) | 0.973 | 0.314 | <0.001(HS) | <0.001 (HS) | 0.028 (S) |
| Lumbar lordosis (LL) | | | | | | |
| Test‡ | -1.458 | -0.272 | -0.3 | -2.773c | -2.845 | -.487 |
| p-value (Sig.) | 0.145 | 0.786 | 0.764 | 0.006 | 0.004 | 0.626 |
| Slip (%) | | | | | | |
| Test‡ | -4.288 | -4.286 | -4.258 | -2.933c | -3.057 | -2.207 |
| p-value (Sig.) | <0.001 (HS) | <0.001 (HS) | <0.001 (HS) | 0.003 | 0.002 | 0.027 |
| L1-L4 | | | | | | |
| Test‡ | -2.43 | -1.629 | -1.515 | -0.685 | -0.314 | -0.763 |
| p-value (Sig.) | 0.015 | 0.103 | 0.130 | 0.493 | 0.753 | 0.44 |
| L4-S1 | | | | | | |
| Test‡ | -2.903 | -2.802 | -3.33 | -0.375 | -2.1 | -0.963 |
| p-value (Sig.) | 0.004 | 0.005 (S) | 0.001 (S) | 1.000 | 0.036 | 0.020 |
| Segmental Lordosis (SL) | | | | | | |
| Test‡ | -4.288 | -3.83 | -3.687 | -3.619 | -3.25 | -3.367 |
| p-value (Sig.) | <0.001(HS) | <0.001(HS) | <0.001(HS) | <0.001 (HS) | <0.001 (HS) | 0.002(S) |
| *Paired t-test, ‡ Wilcoxon signed ranks test, p< 0.05 is significant, Sig.: Significance. | | | | | | |

Appendix Table S2. The comparison according to the SDSG grade

| Parameters | PI | | Test§ | p-value(Sig.) |
| --- | --- | --- | --- | --- |
|  | SDSG2 (N=9) | SDSG3 (n=15) |  |  |
| Δ Pelvic Tilt (PT) | | | | |
| Mean ± SD | 1.37 ± 5.20 | -2.79 ± 5.66 | -2.001 | 0.045(S) |
| Median (Range) | 2.30 (-10.70 – 5.70) | -4.80 (-10.60 – 9) |  |  |
| Δ Sacral Slope (SS) | | | | |
| Mean ± SD | -1.56 ± 5.56 | 2.94 ± 5.70 | -1.822 | 0.068 |
| Median (Range) | -2 (-6.50 – 10.80) | 4.80 (-9 – 10.50) |  |  |
| Δ Lumbar lordosis (LL) | | | | |
| Mean ± SD | -0.62 ± 5.86 | 1.50 ± 10.17 | -0.508 | 0.612 |
| Median (Range) | -0.90 (-10 – 9) | -0.10 (-13 – 14.60) |  |  |
| ΔMismatch | | | | |
| Mean ± SD | -1.44 ± 5.52 | -5.72 ± 7.20 | -1.403 | 0.160 |
| Median (Range) | -0.30 (-10.80 – 6.10) | -7.70 (-14.50 – 6.10) |  |  |
| ΔL1-L4 | | | | |
| Mean ± SD | -1.56 ± 3.31 | -2.96 ± 7.78 | -1.165 | 0.244 |
| Median (Range) | -2.20 (-5.60 – 3.60) | -6 (-12.30 – 7.80) |  |  |
| ΔL4-S1 | | | | |
| Mean ± SD | 2.62 ± 4.12 | 4.53 ± 4.29 | -0.925 | 0.355 |
| Median (Range) | 3.90 (-5 – 9) | 6.70 (-3 – 10.20) |  |  |
| Δ Segmental Lordosis (SL) | | | | |
| Mean ± SD | 3.07 ± 2.67 | 5.18 ± 4.49 | -1.492 | 0.136 |
| Median (Range) | 3.90 (-0.90 – 7.40) | 4.70 (-6.20 – 12.20) |  |  |
| Δ VAS: VAS baseline – VAS final, Δ ODI: ODI baseline – ODI final, Δ PT: PT final – PT baseline, Δ SS: SS final – SS baseline, Δ LL: LL final – LL baseline, Δ LL: LL final – LL baseline, Δ L1-L4: L1-L4 final – L1-L4 baseline, Δ Mis-match: Mis-match final – Mis-match baseline, Δ SL: SL final – SL baseline, § Mann Whitney U test, p< 0.05 is significant and Sig.: Significance | | | | |
