# Supplementary material for: Does minimally invasive transforaminal lumbar interbody fusion (MIS-TLIF) influence functional outcomes and spinopelvic parameters in isthmic spondylolisthesis?
Source: J Orthop Surg Res. 2022 May 15;17:272. doi: 10.1186/s13018-022-03144-y (PMC9107691; doi:10.1186/s13018-022-03144-y)

**Appendix (case 2) Fig. 1** shows (a) preoperative long-standing x-ray with spinopelvic parameters measurments, (b) preoperative MRI showing L5-S1 Isthmic Spondylolisthesis, (c) postoperative long-standing x-ray with spinopelvic parameters measurments, and (d) postoperative follow-up at one year follow up CT shows nonunion.


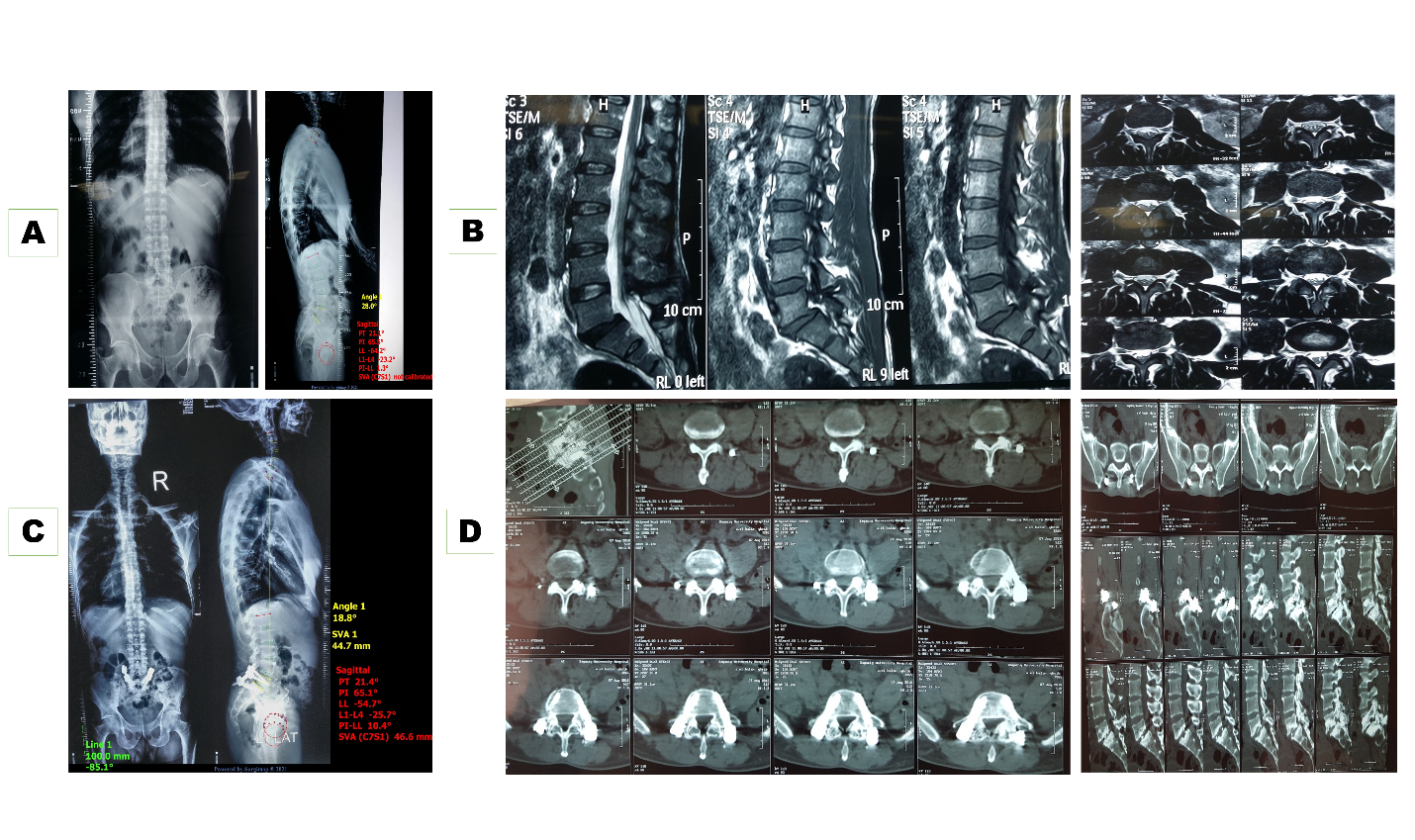

Supplement: Supplementary file 2 — Additional file 2: Figure S1 (case 2) shows (a) preoperative long-standing X-ray with spinopelvic parameters measurements, (b) preoperative MRI showing L5–S1 isthmic spondylolisthesis, (c) postoperative long-standing X-ray with spinopelvic parameters measurements, and (d) postoperative follow-up at 1-year follow-up CT shows nonunion. [file 13018_2022_3144_MOESM2_ESM.docx]
