# Supplementary material for: Does minimally invasive transforaminal lumbar interbody fusion (MIS-TLIF) influence functional outcomes and spinopelvic parameters in isthmic spondylolisthesis?
Source: J Orthop Surg Res. 2022 May 15;17:272. doi: 10.1186/s13018-022-03144-y (PMC9107691; doi:10.1186/s13018-022-03144-y)

**Appendix (case -3) Fig. 3** shows under C-arm guide inseration of screws in AP and lateral view, intraoperative photo of the wound and the tube retractor tube, wound size after closure.


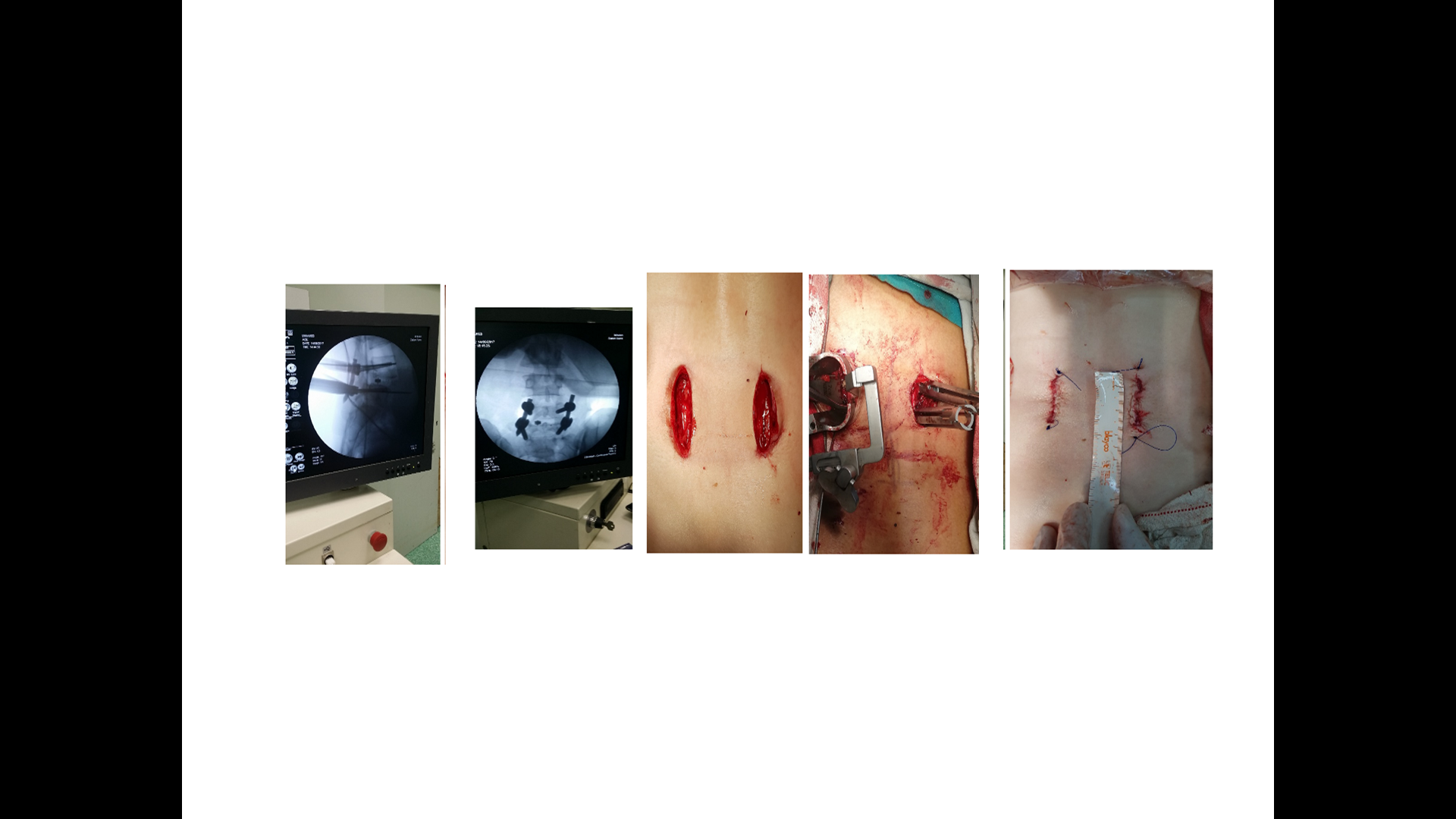

Supplement: Supplementary file 4 — Additional file 4: Figure S3 (case 3) shows under C-arm-guided insertion of screws in AP and lateral view, intraoperative photograph of the wound and the tube retractor tube, wound size after closure. [file 13018_2022_3144_MOESM4_ESM.docx]
